# A Durable Porcine Pericardial Surgical Bioprosthetic Heart Valve: a Proof of Concept

**DOI:** 10.1007/s12265-019-09868-3

**Published:** 2019-02-12

**Authors:** Benyamin Rahmani, Christopher McGregor, Guerard Byrne, Gaetano Burriesci

**Affiliations:** 1 Cardiovascular Engineering Laboratory, UCL Mechanical Engineering, University College London, Torrington Place, London, WC1E 7JE UK; 2 Institute of Cardiovascular Science, University College London, London, UK; 3 Department of Surgery, University of Alabama, Birmingham, AL USA; 4 Ri.MED Foundation, Bioengineering Group, Palermo, Italy

**Keywords:** Biological heart valve, Porcine pericardium, Xenotransplantation, Gal knockout, Calcification

## Abstract

Bioprosthetic leaflets made from animal tissues are used in the majority of surgical and transcatheter cardiac valve replacements. This study develops a new surgical bioprosthesis, using porcine pericardial leaflets. Porcine pericardium was obtained from genetically engineered pigs with a mutation in the GGTA-1 gene (GTKO) and fixed in 0.6% glutaraldehyde, and used to develop a new surgical valve design. The valves underwent in vitro hydrodynamic test in a pulse duplicator and high-cycled accelerated wear testing and were evaluated for acute haemodynamics and thrombogenicity in a juvenile sheep implant study for 48 h. The porcine surgical pericardial heart valves (pSPHVs) exhibited excellent hydrodynamics and reached 200 million cycles of in vitro durability, with no observable damage. Juvenile sheep implants demonstrated normal valve function with no acute thrombogenic response for either material. The pSPHV incorporates a minimalistic construction method using a tissue-to-tissue design to cover the stent. This new design is a proof of concept alternative to the use of bovine pericardium and synthetic fabric in surgical bioprosthetic heart valves.

## Introduction

Surgical bioprosthetic heart valves (BHVs) have been the gold standard treatment for patients suffering from severe cardiac valve diseases. Contrary to mechanical heart valves (MHVs), BHVs do not require long-term anticoagulation therapy which is associated with high risk of thromboembolic complications and haemorrhage [1]. BHVs have been an effective therapy in older patients for decades, but are prone to age-dependent structural valve degeneration (SVD) and limited durability in patients younger than 60 years old. SVD is associated with leaflet calcification, fibrosis, and tearing [2, 3] which lead to irreversible valve deterioration and haemodynamic disruption [4]. Despite these limitations, the use of BHVs is increasing compared with MHVs [5–9].

BHVs have been typically made by sewing glutaraldehyde-fixed bovine pericardium or porcine aortic valve tissue on a fabric covered polymeric or metal stent. The choice of either bovine pericardium or porcine valve leaflets to construct BHV leaflets had remained unchanged until the introduction of percutaneous transcatheter aortic valve implantation (TAVI) in the early 2000s. The original Cribier designed percutaneous heart valve device utilised equine pericardium for the valve leaflet material. Subsequently, the Cribier-Edwards valve was re-engineered with bovine pericardial leaflets for a first in man study [10].

The use of porcine pericardium as heart valve leaflet material is currently limited to percutaneously implantable valves, where its relatively low thickness is advantageous, helping reduce the delivery system profile. Porcine pericardial leaflet has been exploited by the Medtronic CoreValve and CoreValve Evolut™ R [11], Boston Scientific ACURATE neo™ [12, 13], Abbott Portico™ [14], JenaValve™ [15], and Colibri heart valve (dry porcine pericardium) [16]. Although there is no clinically adopted surgical BHV with porcine pericardial leaflets, the success of porcine pericardial leaflet in TAVI devices, at least for midterm durability, suggests that under the right conditions there is no inherent biophysical prohibition to using this material for heart valve leaflet, even in a surgical BHV. Direct biophysical comparisons between porcine and bovine pericardium have been reported [17] and show that porcine pericardium is thinner, stiffer, and less extensible compared with adult or calf bovine pericardium, but maintains similar ultimate tensile strength. The use of thinner leaflet materials in a surgical replacement heart valve, if durable, would be expected to increase the effective orifice area and improve hydrodynamic function.

There is growing interest in using Gal-free porcine tissue as BHV material because elimination of the Gal antigen may reduce antibody associated BHV calcification, especially in younger patients [18–22]. We have previously reported biochemical and physical equivalence of glutaraldehyde-fixed porcine pericardium derived from standard pigs and from pigs with an engineered mutation in the GGTA1 gene to block expression of the galactose α 1,3 galactose (Gal) antigen (GTKO pigs) [23]. Both types of glutaraldehyde-fixed pericardium demonstrated the same general morphology and collagen content and no significant differences in uniaxial stress or suture retention testing. In this paper, we describe and evaluate the design of a new porcine surgical pericardial heart valve (pSPHV) and test its hydrodynamics and in vitro durability. We also perform an acute, 48 h, in vivo study in juvenile sheep to assess the valve function and thrombogenicity.

## Methods

### Pig Tissue Processing and Fixation

Standard pig tissue was obtained from a local abattoir. Genetically modified GGTA-1 deficient (GTKO) pigs were bred and raised in accordance with the Animals (Scientific Procedures) Act of 1986, published by the UK Home Office, and the Guide for the Care and Use of Laboratory Animals from the US National Institute of Health (Publication No. 85-23, revised 1996). Heart lung blocks were collected from pigs, chilled on ice, and promptly shipped for processing. On receipt, the tissues were rinsed in water and dissected to recover the pericardial sac. The pericardium was rinsed in sterile saline, split, grossly cleared of fat, trimmed, washed thoroughly in sterile saline, and stored overnight at 4 °C. The pericardium was subsequently carefully cleaned of fat and fixed in 0.6% glutaraldehyde in 20 mM HEPES-Saline (pH 7.4) containing 13 mM MgCl_2_-H_2_O for 24 h at 4 °C. After fixation, tissues were rinsed in sterile saline and stored in fixation buffer containing 0.2% glutaraldehyde at 4 °C until used.

### Valve Construction

**Fig. 1:**
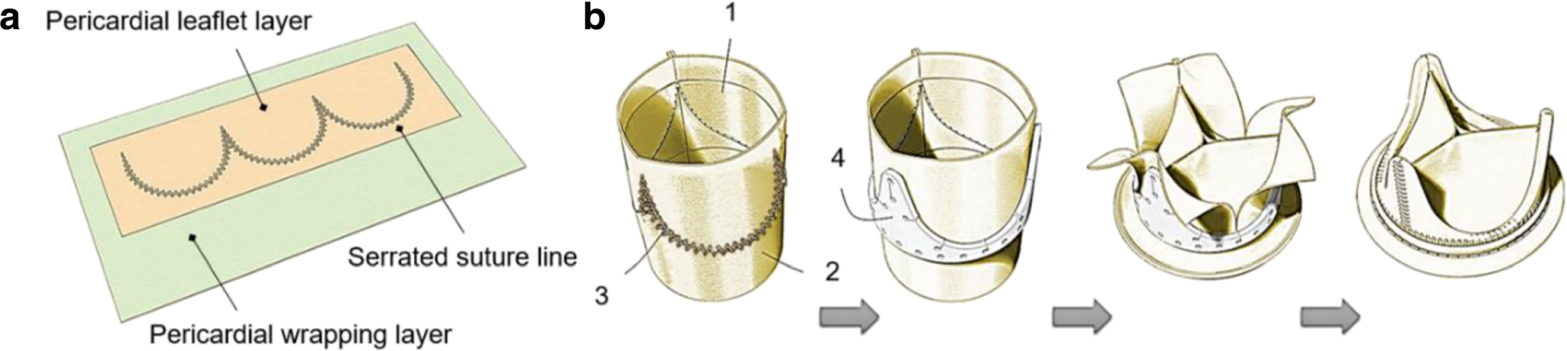
Schematic diagram of **a** the arrangement of pericardial layers and leaflets suture line, and **b** construction steps used for the new bioprosthetic heart valve, demonstrating the pericardial leaflets (1); pericardial wrapping layer (2); leaflet suture line (3); Delrin valve frame (4)

The construction technique adopted a new approach [24] where the pSPHV is formed with three leaflets from a single continuous strip of pericardium, attached to a second pericardial layer which wraps around a Delrin stent of 25 mm internal diameter and 1.2 mm of wall thickness (Fig. 1). Pericardial tissues were quality controlled by measuring and recording the thickness distribution of the glutaraldehyde-fixed patches using a Mitutoyo thickness gauge. Tissue fibre density and orientation were mapped by observation on a viewbox. Pericardial patches for leaflet construction were selected based on this visual assessment and the tissue thickness. Pericardium covering the stent was similarly selected for homogeneity and the absence of visual defects. The pSPHV was constructed by superposing and fixing together the two layers of pericardium by automate sewing of a serrated suture line defining the tri-leaflets profile (Fig. 1a). The two pericardial layers were rolled and closed on the side to form a tubular assembly, which was then mounted on the stent. The support pericardial layer was cut, wrapped around the stent posts, and sewn to the downstream edge of the same pericardial layer to fully cover the valve stent [25] (Fig. 1b). No leaflet pressure fixation was used during the construction process. One pSPHV was prepared for the purpose of in vitro assessment. Two GTKO pSPHVs were constructed for acute evaluation in a juvenile sheep model.

### In Vitro Hydrodynamic Assessment

The in vitro hydrodynamic performance of the valve was evaluated using a commercial hydromechanical pulse duplicator (Vivitro Superpump System SP3891, Vivitro, Victoria, BC, Canada), reproducing physiologically equivalent aortic pressures and flows, in conformity to the international standard ISO5840 requirements [26]. The valve was mounted on the aortic test chamber and tested at increasing cardiac outputs (COs) of 2, 3, 4, 5, 6, and 7 l/min, with a mean arterial pressure of 100 mmHg, a fixed heart rate of 70 beats per minute, and systole occupying 35% of the cardiac cycle. Testing fluid was a 37 °C buffered saline solution (0.90% *w*/*v* NaCl).

Under stabile mean arterial pressure and the cardiac output flow, measurements of atrial, ventricular, and aortic pressures and aortic flow were collected during 10 consecutive cardiac cycles, using the ViViTest software. Based on these recordings, the mean transvalvular systolic pressure drop (∆*P*), the total regurgitant volume, and the regurgitant fraction were calculated. The total regurgitant volume includes the closing regurgitant volume, associated with the dynamic of valve closure, and the leakage regurgitant volume, corresponding to the leakage through the closed valve. The total regurgitant fraction represents the regurgitant volume expressed as a percentage of the stroke volume. Effective orifice area (EOA), which represents the minimal cross-sectional area of the downstream jet emerging from aortic valve orifice [27], was derived from the continuity equation, applying Gorlin’s formula [28].

### Accelerated Wear Test

The functional durability of the valve design was evaluated in an in vitro accelerated wear test (AWT) setup, using a VDT-3600i AWT system (BDC Laboratories, CO, USA). The valve was mounted on a test chamber, with 37 ± 1 °C buffered saline with 1 g/l of sodium azide testing fluid as a fungicide and bactericide, running at 10 Hz cycle rate, set to maintain a peak differential pressure above 100 mmHg across the closed valve for at least 5% of each cycle [26]. The system software provides continual monitoring of the real-time differential pressures, recording only the cycles where pressure conditions complied with the specified testing requirements. The test was initially run for 200 million cycles, as requested by the ISO5840 standard for flexible leaflet heart valve substitutes. The valve was visually inspected for any signs of damage on daily basis during working days, and functionally evaluated in the pulse duplicator before and after completing the 200 million cycles.

### Acute Animal Evaluation

Valves GTKO pericardium based on the pSPHV design (*n* = 2) were implanted in female juvenile sheep for 48 h, in the orthotopic mitral position. The implants were performed at a GLP-compliant preclinical research centre, Institut Mutualiste Montsouris Recherche (IMMR, Paris, France), in accordance with the Animals (Scientific Procedures) Act 1986. Post-implant transthoracic echocardiography was performed to assess the valve haemodynamics. At explant (48 h), the animals were sacrificed humanely by intravenous overdose of barbiturate, and the heart was explanted and dissected.

## Results

**Fig. 2:**
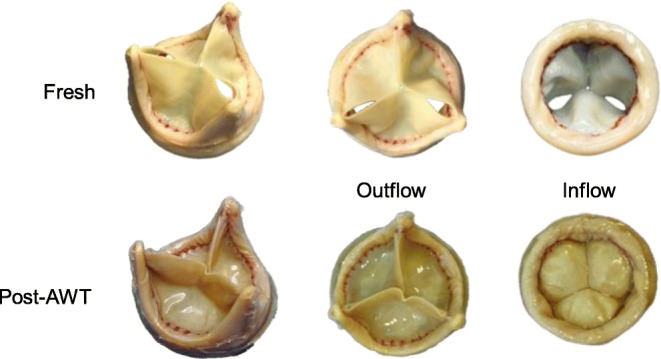
GTKO porcine pericardial bioprosthesis, top row: newly made before undergoing AWT, and bottom row: after 200 million cycles of AWT. Post-AWT, the pericardium was slightly discoloured, with no visible damage, and the leaflets were deformed into a trifoil shapes (as happens following pericardium pressure fixation). The serrated leaflet suture line is visible in red colour

**Fig. 3:**
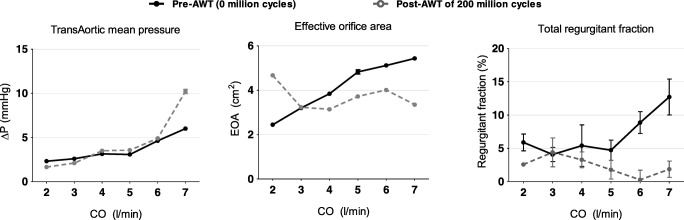
Hydrodynamic test parameters evaluated over increasing cardiac output. Left: aortic pressure gradient (∆*P*), centre: effective orifice area (EOA), right: percent regurgitant fraction. Data are presented as means ± standard deviation. Solid black lines represent the valve performance prior to undergoing high-cycled AWT, and dashed grey lines represent the data after 200 million cycles of AWT

The pSPHV was successfully manufactured, with an average leaflet thickness of 0.35 ± 0.4 mm and a pericardial stent covering of 1.8 ± 0.1 mm thickness (Fig. 2). The valve was assessed for its hydrodynamic function prior to undergoing high-cycled AWT. As the cardiac output in the pulse duplicator increased from 2 to 7 l/min, the valve demonstrated a systolic pressure drop rising from 2.32 ± 0.05 to 6.00 ± 0.16 mmHg and an effective orifice area enlarging from 2.44 ± 0.02 to 5.43 ± 0.06 cm^2^. The regurgitant fraction was stable below 6% up to 5 l/min, then linearly increased to 12.71 ± 2.70% at 7 l/min (solid lines in Fig. 3).

AWT was used to assess the durability of the pSPHV. At a pulse frequency of 10 Hz, the valve remained stable, maintaining the set peak differential pressure of ≥ 100 mmHg across the closed valve for at least 5% of each cycle, as prescribed by the international standard ISO5840 [26]. After completing 200 million cycles, the valve was removed from the AWT rig and showed no sign of structural damage at visual inspection. The hydrodynamic performance of the pSPHV demonstrated a comparable pre- and post-AWT pressure drops at cardiac outputs up to 6 l/min. The post-AWT pressure drop was higher (10.21 ± 0.24 mmHg) at 7 l/min. The effective orifice area was maintained in the range from 3.35 ± 0.05 to 4.67 ± 0.03 cm^2^ at different output conditions. The regurgitant fraction was reduced significantly and remained below 4.39 ± 2.17 throughout all cardiac outputs (dashed lines in Fig. 3).

**Fig. 4:**
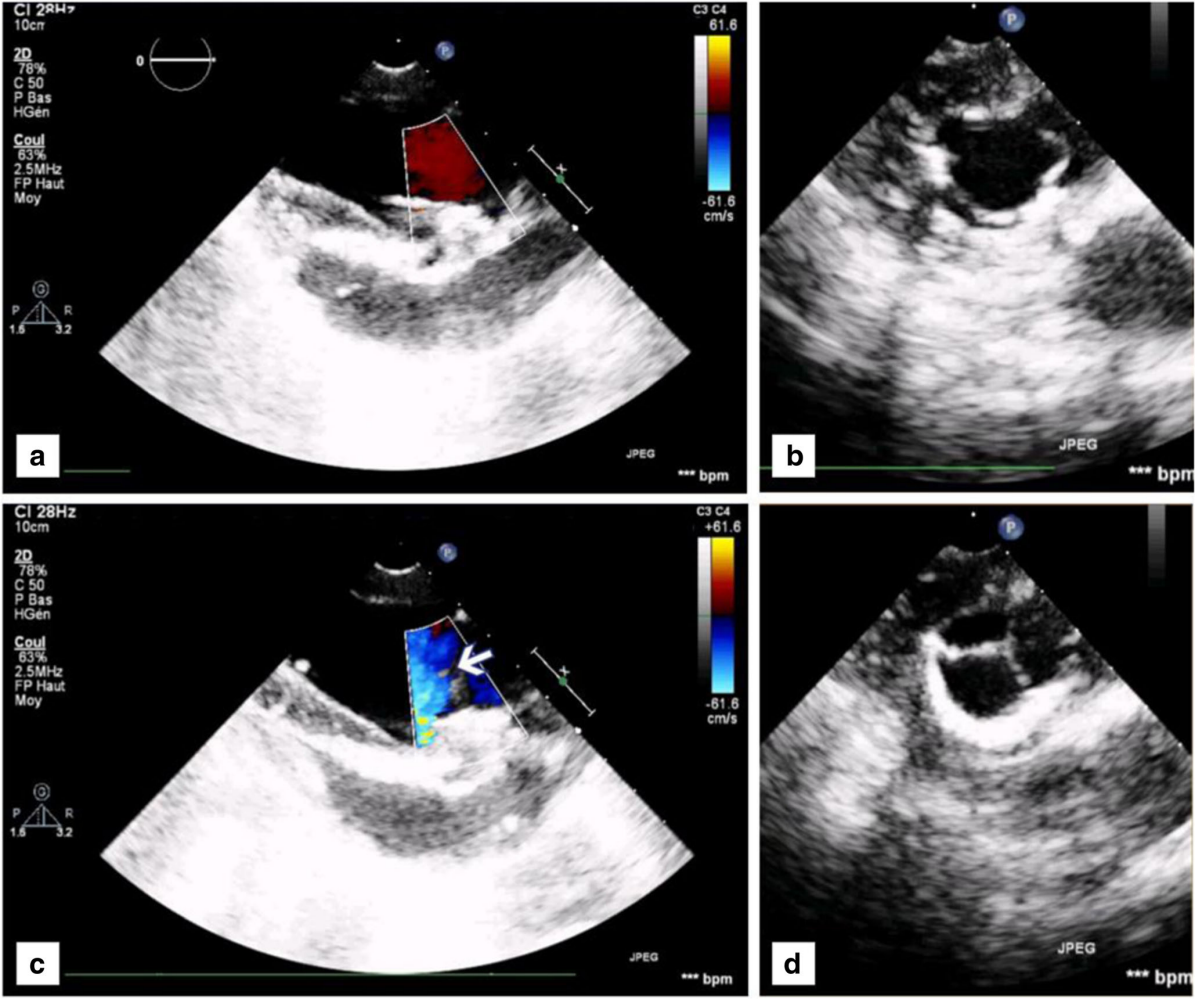
Echo-Doppler images illustrating the haemodynamics performances of GTKO pSVHV showing **a**, **b** full opening and laminar flow through the leaflets and **c**, **d** full coaptation and competence of the leaflets

The pSPHVs were successfully implanted in the ovine model. Continuous wave Doppler examination immediately after implantation and prior to explant showed good haemodynamics with low transvalvular gradients. The valves displayed full opening of the leaflets with laminar flow through a wide orifice area (Fig. 4a, b), and full coaptation and competence of the leaflets during systole, with no measurable leakage (Fig. 4c, d). The mean transvalvular pressure gradient measured as 3 mmHg, reaching a maximum of 6 mmHg. The mean and maximum jet velocities were measured as 74.0 and 122 cm/s, respectively. The velocity integral was 19.8 cm. On explant, there was no evidence of valve induced thrombosis.

## Discussion

In this study, we investigated a new surgical bioposthetic valve design using porcine pericardium as the leaflet material. The leaflets were formed from a single patch of pericardium, attached to a second pericardial layer (support layer) to connect the leaflets to the frame (Fig. 1). This approach minimises the overall valve profile by allowing the entire inner and outer surfaces of the valve, including the sewing cuff, to be covered by no more than a single layer of pericardium. It also provides tissue-to-tissue contact of the pericardial leaflets to the pericardial-covered frame, minimising the risk of leaflet abrasion and related structural deterioration. The in vitro hydrodynamic performance of the pSPHV met and exceeded the minimum requirements of ISO5840 for both aortic and mitral applications. The ISO standard specifies the performance requirements, as a function of valve size, in terms of minimum EOA and maximum regurgitant fraction achieved at a CO of 5 l/min [26]. Assuming a supra-annular implant position, the valve would be suitable for a tissue annulus of 25 mm, requiring a minimum EOA larger than 1.45 cm^2^, and a regurgitant fraction below 15%. In the case of an intra-annular implantation, the most suitable tissue annulus diameter would be of 28 mm, which is not specified in the standard. Considering the more conservative implantation size of 29 mm, the minimum required EOA is equal to 1.95 cm^2^, and the maximum tolerated regurgitant fraction is of 20%. This sets a range of EOA (1.45–1.95 cm^2^) and regurgitant fraction (15–20%) for the minimal performance standard of the pSPHV. At a cardiac output of 5 l/min, the pSPHV design shows an EOA of 4.83 ± 0.10 and 3.72 ± 0.0 cm^2^, pre- and post-AWT, and a regurgitant fraction of 4.72 ± 1.52 and 1.78 ± 1.41% pre- and post-AWT, respectively. The pSPHV demonstrated similar pressure drops pre- and post-AWT when tested in the pulse duplicator setup. The unusually higher peak of 10.21 ± 0.24 mmHg at 7 l/min post-AWT is likely to be due to leaflet fluttering. This is a common phenomenon with bioprosthetic heart valves, often reported at high COs [29] and can result in some loss in the valve efficiency. These excellent hydrodynamics were similarly maintained across all tested cardiac outputs (Fig. 3). The pSPHV investigated in this study was durable and successfully passed 200 million cycles of AWT, equivalent to 5 years of normotensive function, as per the international standard [26]. Preliminary studies in juvenile sheep demonstrate that this valve design had normal haemodynamic function (Fig. 4).

While porcine pericardium is used in transcatheter valve designs, including Medtronic CoreValve, Boston Scientific ACURATE neo™, Abbott Portico™, JenaValve™, and Colibri heart valve [11–16], this is the first instance we are aware of using porcine pericardium as leaflet material in a durable surgical heart valve design. Current surgical BHVs made from porcine valve leaflets or bovine pericardium typically have a multi-layered structure with several layers of biocompatible textile and biological tissue sutured to one another. Intricate suturing of multiple layers is a more expensive construction method and may lead to an increase in potential failure points on the implant [30]. Furthermore, having multiple fabric/pericardial layers can compromise the valve’s hydrodynamic performance, as the additional layers tend to reduce the internal diameter of valve, narrowing its orifice area and increasing the resultant transvalvular pressure drop for a given nominal size [31]. Porcine pericardial leaflets, being 30–40% thinner than their bovine counterparts, allow for valves with more flexible leaflets which can attain wider orifice areas, thus improving haemodynamics. The use of porcine pericardium is largely confined to applications which benefit from the smaller thickness such as minimally invasive BHV replacement and percutaneous bioprostheses. In fact, the reduction in prosthetic profile and crimp diameter enabled by the thinner tissue represent a major advantage, simplifying advancement of the catheter through smaller arteries and reducing the associated risk of vascular complication. Currently, a number of transcatheter valves, such as the CoreValve System by Medtronic (Minneapolis, MN) and the ACURATE neo valve by Boston Scientific (Marlborough, MA), employ porcine pericardial leaflets as an alternative to bovine pericardium. These devices have shown a successful midterm outcome in inoperable and high-risk patients, when compared with surgical bioprosthesis [32–36]. Although the thinner porcine pericardial tissue is necessarily structurally weaker than its bovine alternative, this study suggests that a durable surgical heart valve using porcine pericardial leaflets can also be produced.

There is growing interest in using Gal-free animal tissues from Gal-knockout pigs (GTKO) to reduce anti-Gal antibody-associated tissue calcification [18, 21, 22, 37, 38]. While the potential advantage of Gal-free tissue has not been clinically demonstrated, our previous studies have shown that glutaraldehyde-fixed GTKO and standard porcine pericardial tissues have same general morphology, collagen content, and mechanical strength [23]. In this study, we showed that the new pSPHV design made with GTKO pericardium exhibited normal haemodynamic function and no evidence of unusual thrombogenicity in an acute, 48 h, juvenile sheep study. Further long-term animal studies are required to establish equal biological function. This study was limited by the number of available valves. A more detailed investigation using this same valve design, but with larger sample number, is currently ongoing to further evaluate the hydrodynamic performance and durability, and to compare the in vivo biological performance and safety of valves made from standard and GTKO pericardium. The current study suggests that porcine pericardium has adequate structural strength to be used as surgical BHV leaflets material, in the context of a valve design which incorporates a minimal overall valve profile and tissue-to-tissue contact of the pericardial leaflets to the pericardial-covered frame. The new pSPHV design developed in this study was durable and showed excellent haemodynamics, supporting the feasibility of using thinner leaflet materials in surgical heart valves.

## Conclusion

A novel surgical bioprosthesis using porcine pericardial leaflets, an improved design and a refined construction technique, was developed and assessed. The new minimalistic construction method allows for making a bioprosthesis with smaller profile, and thinner leaflets, which can be durable reaching 200 million cycles of AWT with no visible damage, and exhibits excellent hydrodynamics. These encouraging results suggest that durable surgical valves with improved hydrodynamics can be produced using thinner porcine pericardial leaflets.

## Abbreviations

AWT: Accelerated wear test
BHV: Bioprosthetic heart valve
CO: Cardiac output
EOA: Effective orifice area
GGTA-1: Alpha-1,3-galactosyltransferase
GLP: Good laboratory practice
GTKO: α-Galactosyltransferase knockout
ISO: International Standard Organization
MHV: Mechanical heart valve
pSPHV: Porcine surgical pericardial heart valve
SVD: Structural valve degeneration
TAVI: Transcatheter aortic valve implantation

## References

[1] Goldstone AB, Chiu P, Baiocchi M, Lingala B, Patrick WL, Fischbein MP, Woo YJ (2017). Mechanical or biologic prostheses for aortic-valve and mitral-valve replacement. The New England Journal of Medicine.

[2] Dvir, D., Bourguignon, T., Otto, C. M., Hahn, R. T., Rosenhek, R., Webb, J. G., et al. (2018). Standardized definition of structural valve degeneration for surgical and transcatheter bioprosthetic aortic valves. *Circulation*, 388–399. 10.1161/CIRCULATIONAHA.117.030729 29358344

[3] Capodanno, D., Petronio, A. S., Prendergast, B., Eltchaninoff, H., Vahanian, A., Modine, T., … Haude, M. (2017). Standardized definitions of structural deterioration and valve failure in assessing long-term durability of transcatheter and surgical aortic bioprosthetic valves: a consensus statement from the European Association of Percutaneous Cardiovascular Interven. *European Heart Journal, 38*(45), 3382–3390. 10.1093/eurheartj/ehx303 29020344

[4] Johnston, D. R., Soltesz, E. G., Vakil, N., Rajeswaran, J., Roselli, E. E., Sabik, J. F., … Blackstone, E. H. (2015). Long-term durability of bioprosthetic aortic valves: implications from 12,569 implants. *Annals of Thoracic Surgery, 99*(4), 1239–1247. 10.1016/j.athoracsur.2014.10.070 PMC5132179 25662439

[5] Dunning, J., Gao, H., Chambers, J., Moat, N., Roxburgh, J., Cth, F., … Cth, F. (2011). Aortic valve surgery: marked increases in volume and significant decreases in mechanical valve use — an analysis of 41 , 227 patients over 5 years from the Society for Cardiothoracic Surgery in Great Britain and Ireland National database. *The Journal of Thoracic and Cardiovascular Surgery, 142*(4), 776–782.e3. 10.1016/j.jtcvs.2011.04.048 21924147

[6] Isaacs AJ, Shuhaiber J, Salemi A, Isom OW, Sedrakyan A (2015). National trends in utilization and in-hospital outcomes of mechanical versus bioprosthetic aortic valve replacements. Journal of Thoracic and Cardiovascular Surgery.

[7] Zhao, D. F., Seco, M., Wu, J. J., Edelman, J. B., Wilson, M. K., Vallely, M. P., … Bannon, P. G. (2016). Mechanical versus bioprosthetic aortic valve replacement in middle-aged adults: a systematic review and meta-analysis. *Annals of Thoracic Surgery, 102*(1), 315–327. 10.1016/j.athoracsur.2015.10.092 26794881

[8] Kappetein SJHMÇAP (2017). Clinical update Mechanical versus bioprosthetic aortic valve replacement. European Heart Journal.

[9] Wang Y, Chen S, Shi J, Li G, Dong N (2016). Mid-to long-term outcome comparison of the Medtronic Hancock II and bi-leaflet mechanical aortic valve replacement in patients younger than 60 years of age: a propensity-matched analysis. Interactive Cardiovascular and Thoracic Surgery.

[10] Cribier, A., Eltchaninoff, H., Bash, A., Borenstein, N., Tron, C., Bauer, F., … Leon, M. B. (2002). Percutaneous transcatheter implantation of an aortic valve prosthesis for calcific aortic stenosis: first human case description. *Circulation, 106*(1524–4539), 3006–3008. 10.1161/01.cir.0000047200.36165.b8 12473543

[11] Grube, E., Laborde, J. C., Gerckens, U., Felderhoff, T., Sauren, B., Buellesfeld, L., … Stone, G. W. (2006). Percutaneous implantation of the CoreValve self-expanding valve prosthesis in high-risk patients with aortic valve disease: the Siegburg first-in-man study. *Circulation, 114*(15), 1616–1624. 10.1161/CIRCULATIONAHA.106.639450 17015786

[12] Mauri, V., Kim, W. K., Abumayyaleh, M., Walther, T., Moellmann, H., Schaefer, U., … Rudolph, T. K. (2017). Short-term outcome and hemodynamic performance of next-generation self-expanding versus balloon-expandable transcatheter aortic valves in patients with small aortic annulus: a multicenter propensity-matched comparison. *Circulation: Cardiovascular Interventions, 10*(10), 1–7. 10.1161/CIRCINTERVENTIONS.117.005013. 10.1161/CIRCINTERVENTIONS.117.005013 28951395

[13] Husser, O., Kim, W. K., Pellegrini, C., Holzamer, A., Walther, T., Mayr, P. N., … Hengstenberg, C. (2017). Multicenter comparison of novel self-expanding versus balloon-expandable transcatheter heart valves. *JACC: Cardiovascular Interventions, 10*(20), 2078–2087. 10.1016/j.jcin.2017.06.026. 10.1016/j.jcin.2017.06.026 29050625

[14] Manoharan G, Linke A, Moellmann H, Redwood S, Frerker C, Kovac J, Walther T (2016). Multicentre clinical study evaluating a novel resheathable annular functioning self-expanding transcatheter aortic valve system: safety and performance results at 30 days with the Portico system. EuroIntervention.

[15] Silaschi, M., Treede, H., Rastan, A. J., Baumbach, H., Beyersdorfe, F., Kappert, U., … Wendler, O. (2016). The JUPITER registry: 1-year results of transapical aortic valve implantation using a second-generation transcatheter heart valve in patients with aortic stenosis. *European Journal of Cardio-Thoracic Surgery, 50*(5), 874–881. 10.1093/ejcts/ezw170. 10.1093/ejcts/ezw170 27242354

[16] Fish RD, Paniagua D, Ureña P, Chevalier B (2013). The Colibri heart valve: theory and practice in the achievement of a low-profile, pre-mounted, pre-packaged TAVI valve. EuroIntervention.

[17] Caballero A, Sulejmani F, Martin C, Pham T, Sun W (2017). Evaluation of transcatheter heart valve biomaterials: biomechanical characterization of bovine and porcine pericardium. Journal of the Mechanical Behavior of Biomedical Materials.

[18] Zhang R, Wang Y, Chen L, Wang R, Li C, Li X (2018). Reducing immunoreactivity of porcine bioprosthetic heart valves by genetically-deleting three major glycan antigens, GGTA1/β4GalNT2/CMAH. Acta Biomaterialia.

[19] Konakci, K. Z., Bohle, B., Blumer, R., Hoetzenecker, W., Roth, G., Moser, B., … Ankersmit, H. J. (2005). Alpha-Gal on bioprostheses : xenograft immune response in cardiac surgery. *European Journal of Clinical Investigation, 35*, 17–23. 10.1111/j.1365-2362.2005.01441.x 15638815

[20] Park CS, Park SS, Choi SY, Yoon SH, Kim WH, Kim YJ (2010). Anti alpha-Gal immune response following porcine bioprosthesis implantation in children. The Journal of Heart Valve Disease.

[21] McGregor CG, Carpentier A, Lila N, Logan JS, Byrne GW (2011). Cardiac xenotransplantation technology provides materials for improved bioprosthetic heart valves. The Journal of Thoracic and Cardiovascular Surgery.

[22] Lila N, McGregor CG a, Carpentier S, Rancic J, Byrne GW, Carpentier A (2010). Gal knockout pig pericardium: new source of material for heart valve bioprostheses. Journal of Heart and Lung Transplantation.

[23] McGregor C, Byrne G, Rahmani B, Chisari E, Kyriakopoulou K, Burriesci G (2016). Physical equivalency of wild type and galactose α 1,3 galactose free porcine pericardium; a new source material for bioprosthetic heart valves. Acta Biomaterialia.

[24] Burriesci, G., Rahmani, B., McGregor, C., & Byrne, G. (2018). Prosthetic heart valve. WO2018011592.

[25] Burriesci, G., Rahmani, B. (2016). Design application No: 003002088-0001.

[26] ISO 5840-2. (2015). Cardiovascular implants. Cardiac valve prostheses. In *Surgically implanted heart valve substitutes*. Geneva: ISO.

[27] Garcia D, Kadem L (2006). What do you mean by aortic valve area: geometric orifice area, effective orifice area, or gorlin area?. The Journal of Heart Valve Disease.

[28] Gorlin R, Gorlin SG (1951). Hydraulic formula for calculation of the area of the stenotic mitral valve, other cardiac valves, and central circulatory shunts. American Heart Journal.

[29] Avelar AHF, Canestri JA, Bim C, Silva MGM, Huebner R, Pinotti M (2017). Quantification and analysis of leaflet flutter on biological prosthetic cardiac valves. Artificial Organs.

[30] Siddiqui RF, Abraham JR, Butany J (2009). Bioprosthetic heart valves: modes of failure. Histopathology.

[31] Tasca, G., Fiore, G. B., Redaelli, P., Romagnoni, C., Redaelli, A., Gamba, A., … Vismara, R. (2017). Hydrodynamic and geometric behavior of two pericardial prostheses implanted in small aortic roots. *ASAIO Journal, 64*(1), 86–90. 10.1097/MAT.0000000000000587 28475560

[32] El Faquir, N., Ren, B., Faure, M., de Ronde, M., Geeve, P., Maugenest, A. M., … Van Mieghem, N. M. (2017). Long-term structural integrity and durability of the Medtronic CoreValve System after transcatheter aortic valve replacement. *JACC: Cardiovascular Imaging*, (3). 10.1016/j.jcmg.2017.08.019 29153578

[33] Baron, S. J., Arnold, S. V., Reynolds, M. R., Wang, K., Deeb, M., Reardon, M. J., … Cohen, D. J. (2017). Durability of quality of life benefits of transcatheter aortic valve replacement: long-term results from the CoreValve US extreme risk trial. *American Heart Journal, 194*, 39–48. 10.1016/j.ahj.2017.08.006 PMC5821894 29223434

[34] Barbanti, M., Petronio, A. S., Ettori, F., Latib, A., Bedogni, F., De Marco, F., … Tamburino, C. (2015). 5-year outcomes after transcatheter aortic valve implantation with CoreValve prosthesis. *JACC: Cardiovascular Interventions, 8*(8), 1084–1091. 10.1016/j.jcin.2015.03.024 26117458

[35] Sulzenko J, Tousek P, Kocka V, Widimsky P (2015). Transcatheter aortic valve implantation: long-term clinical outcome and valve durability. Expert Review of Medical Devices.

[36] Arsalan M, Walther T (2016). Durability of prostheses for transcatheter aortic valve implantation. Nature Reviews Cardiology.

[37] Naso, F., Gandaglia, A., Bottio, T., Tarzia, V., Nottle, M. B., D’Apice, A. J. F., … Gerosa, G. (2013). First quantification of alpha-Gal epitope in current glutaraldehyde-fixed heart valve bioprostheses. *Xenotransplantation, 20*(November 2012), 252–261. 10.1111/xen.12044 23865597

[38] Naso F, Stefanelli U, Buratto E, Lazzari G, Perota A, Galli C, Gandaglia A (2017). Alpha-Gal inactivated heart valve bioprostheses exhibit an anti-calcification propensity similar to knockout tissues. Tissue Engineering Part A.

